# A new notable compression source of left renal vein entrapment: right renal artery

**DOI:** 10.1007/s00345-024-05053-7

**Published:** 2024-05-29

**Authors:** Zhanfeng Sun, Haitao Wang, Huijie Jiang, Yongbin Shen, Ziming Shi, Qingxiao Wang, Han Wang, Weiliang Jiang, Xuanyi Du

**Affiliations:** 1 Department of Vascular Surgery, The Second Affiliated Hospital of Harbin Medical University, Harbin, Heilongjiang China; 2 Department of Radiology, The Second Affiliated Hospital of Harbin Medical University, Harbin, Heilongjiang China; 3 Department of Pediatric Orthopaedics, Liaocheng People’s Hospital, Liaocheng, Shandong China; 4 Department of Biostatistics, Kun Tuo Medical Research and Development (Beijing) Co., Ltd, Beijing, China; 5 Department of Nephrology, The Second Affiliated Hospital of Harbin Medical University, 246 Xuefu Road, Nangang District, Harbin, 150086 Heilongjiang China

**Keywords:** Renal nutcracker syndrome, Renal artery, Renal veins, Membranous nephropathy, Peripheral arterial diseases, Computed tomography

## Abstract

**Purpose:**

To estimate the incidences of left renal vein (LRV) entrapment by right renal artery (RRA), a phenomenon primarily reported as case reports.

**Methods:**

The cross-sectional study consecutively screened renal vessel CT data of 38 (Renal) patients with nephropathy and 305 (Non-renal) patients with peripheral arterial diseases in a teaching hospital in northeast China between November 2018 and March 2023. The LRV compression by adjacent anatomical structures, including but not limited to RRA and multiple compression-related parameters, were investigated through multiplanar analysis of the CT data.

**Results:**

The overall LRV entrapment rates by adjacent structures were 41.93% (12/31) and 24.00% (6/25), the rates of RRA-sourced LRV compression 22.58% (7/31) and 20.00% (5/25), and the rates of compression by superior mesenteric artery (SMA) 16.13% (5/31) and 4.00% (1/25) in the Renal and Non-renal groups, respectively, with no significance. The venous segments distal to the RRA-compressed site had a significantly larger transectional lumen area than those of the non-compressed veins in both groups (3.09 ± 1.29 vs. 1.82 ± 0.23, *p* < 0.001 and 4.30 ± 2.65 vs. 2.12 ± 0.55, *p* = 0.006; maximum-to-minimum area ratios in Renal and Non-renal groups, respectively). Nearly 80% of RRAs were found arising anteriorly rightwards instead of passing straight to the right.

**Conclusion:**

RRA-sourced LRV compression was not rare, and its incidence was higher than that of the compression by SMA in both patient cohorts. RRA could be a more common compression source than SMA concerning LRV entrapment. Further investigations involving different populations, including healthy individuals, are needed.

**Supplementary Information:**

The online version contains supplementary material available at 10.1007/s00345-024-05053-7.

## Introduction

Left renal vein (LRV) entrapment is associated with a variety of clinical syndromes, such as hematuria, low back pain, varicocele, and pelvic congestion syndrome [1, 2]. Clinically, LRV entrapment has been almost equated with the nutcracker phenomenon (NCP), in which the compression is due to the course of the superior mesenteric artery (SMA). However, one of the critical challenges of NCP is that the exact NCP prevalence is unknown because of the variability of symptoms and the absence of consensus on the diagnostic criteria [1–3]. The actual incidence of LRV compression may, therefore, be underestimated. Recent literature shows that the incidence of LRV compression can vary from 10.9 to 37.5% in specific populations [4–7].

Many review articles, including a recent systematic review, have shown the insufficient significance of the aortomesenteric angle in diagnosing NCP [1, 2]. The fact that several other indicators must be added to the diagnostic criteria besides the angle value [2, 3] indicates that there might be other latent sources of LRV compression.

The possibility of LRV compression by a right renal artery (RRA) has been validated by a few case reports [8–11]. Meanwhile, there is a markedly higher incidence of left-sided renal venous thrombosis than that of the right side in patients with membranous nephropathy [12–14]. To our knowledge, no reason has been given for such a phenomenon. While exploring the relationship between the NCP and renal venous thrombosis, we inadvertently observed a notable incidence of RRA-sourced LRV compression. This study was designed to investigate this phenomenon by post-processing the CT data of the renal vessels and to test its unrareness in another patient cohort.

## Methods

### Study design

The study screened 38 consecutive hospitalized kidney patients diagnosed with membranous nephropathy by pathological biopsy or antibody test of anti-phospholipase A2 receptor between November 2018 and March 2023. These patients (Renal group) received CT venography of the renal vessels. Three hundred and five consecutive peripheral arterial inpatients or outpatients who underwent abdominal aortic CT angiography examination during the same period were retrospectively selected and screened as another group (Non-renal group). Patients were included in both groups if axial CT imaging could visualize the LRV, SMA, renal arteries, adjacent abdominal aorta, and inferior vena cava (IVC) and was with regular and adequate layer thinness to perform multiplanar reconstruction (MPR). Any potential situations that might cause positional or course changes in proximal RRA or LRV due to pathological reasons were excluded from the study. Detailed exclusion criteria were summarized as Online Resource 1. Parameters concerning LRV compression by different anatomical sources, including but not limited to RRA, were investigated. Other sources of compression other than RRA, were further excluded in the two groups for the subsequent comparisons of relevant variables (Online Resource 1). We used the STROBE cross-sectional checklist when writing our report [15].

### Renal vein CT protocol

Renal vessel imaging in the Renal group was performed using contrast-enhanced spiral CT (Brilliance 256; Philips Medical Systems, Cleveland, OH). All patients underwent craniocaudal scanning in the supine position while holding a single-held breath. A non-ionic contrast agent (Ultrafine, Bayer Schering Pharma AG, German) was injected into the antecubital vein using a dual-cylinder high-pressure syringe at 1.2 ml/kg, 4 ml/s. The venous phase acquisition was set to 80 s after intravenous injection, followed by a flush of 40 mL saline. The scanning range extended from the diaphragmatic roof to the superior border of the pubic symphysis. Scanning parameters were 250 mA at 120 kV, 0.5 mm reconstruction interval, and 0.625 mm increment.

### Image analysis

All CT data post-processing was done by two independent investigators (ZS, HJ) using RadiAnt DICOM Viewer software (version 2021.1, Medixant, Poland). All images were studied both axially and in MPR planes. Measurements were repeated once for all subjects of the study and twice for those with LRV compression. If two RRAs exist, the nearer one to the LRV was studied only. 3-D reconstruction was employed to visualize compressions. Morphological assessment of adjacent segmental abdominal aorta and IVC, surrounding organs, and soft tissue was performed to exclude lesions potentially causing LRV compression, such as masses, aneurysms, or other pathological anomalies.

### Variable definition and measurement

In order to differentiate the LRV compression by different compression sources, the following two conditions should be satisfied simultaneously: (a) The source is in direct contact with LRV and causes a local deformation of the latter; (b) Compared with the adjacent sites, the compressed site of LRV bears a sudden reduction in the transectional area and is close to the compression source, and no other sources can be attributed to the compression. The beak sign, defined as the abrupt narrowing of the LRV lumen between the aorta and the SMA on axial images, or the aortomesenteric angle <41°, measured in adjusted MPR planes was used to detect the SMA compression of LRV [2, 3, 16].

Each LRV was divided into three consecutive segments: The initial segment (Segment 1), from the origin of the main renal vein to the left edge of the aorta; Segment 2, between the aorta and the SMA; and Segment 3, from the right edge of the aorta to its confluence with the IVC. The transectional areas of LRV (perpendicular to the central axis) were measured at three different points in each segment. The maximum and minimum areas were measured at three different points in each segment, the site with the minimum area was categorized, and the maximum-to-minimum area ratio was calculated. The above measurements were repeated twice by two independent investigators (ZS and HJ) and averaged for comparison.

Relationships between LRV and RRA were classified into two subgroups: (a) The Non-compressed subgroup, i.e., with no anatomical border contact of the two vessels or with anatomical border contact but causing no morphological changes in serial observation of transectional imaging, and (b) the Compressed subgroup, meeting the criteria for LRV compression as previously mentioned. Any compressive deformations requiring careful identification were included in the Non-compressed subgroup.

**Fig. 1:**
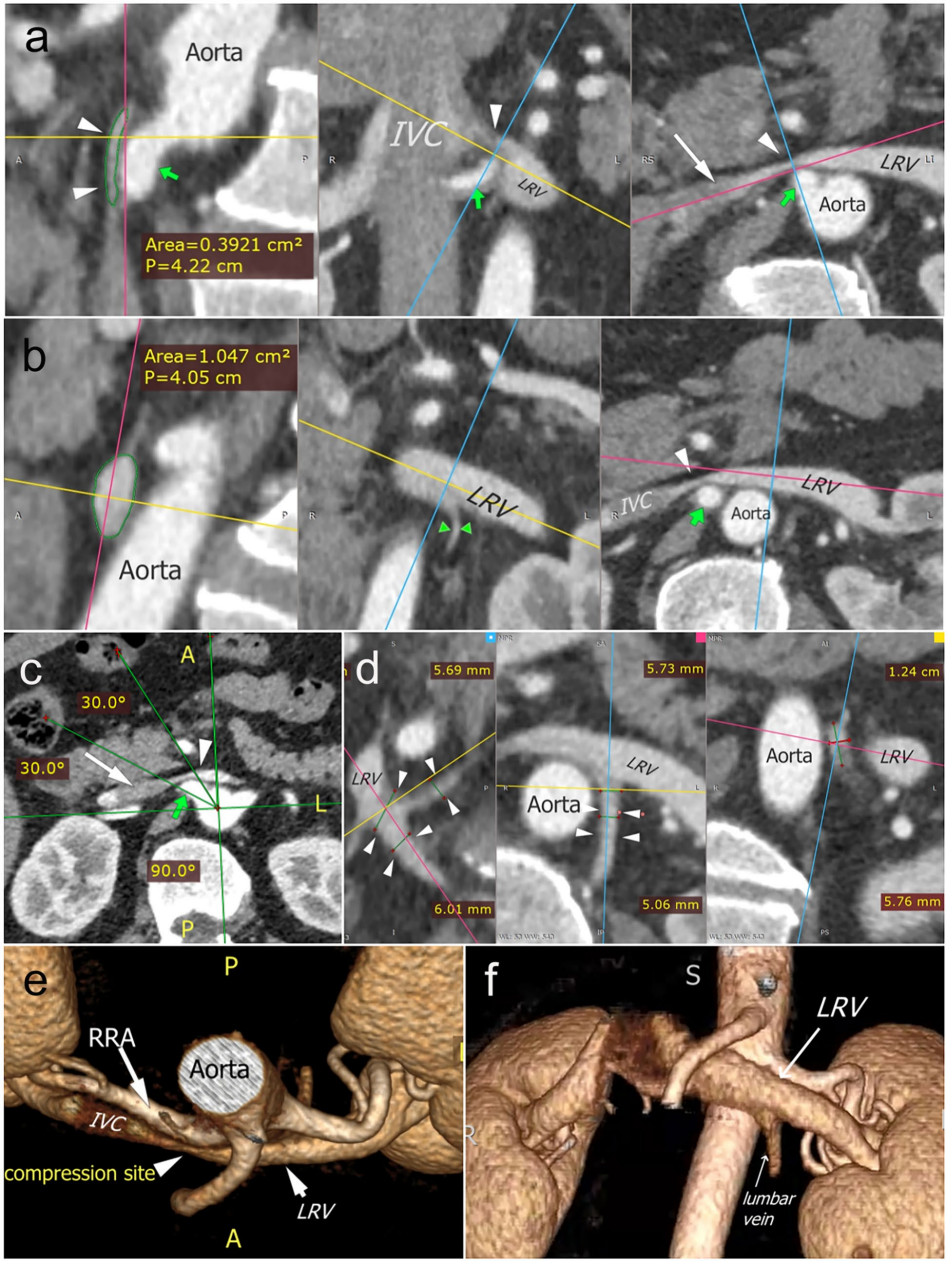
CT imaging showed a notable LRV compression by RRA in a 58-year-old male with membranous nephropathy. The ending segment of LRV was compressed by the proximal RRA (green arrows in **a**–**c**), arising at a 10:30 clockwise direction (**c**). The narrowest site was at the compression site (white arrowheads in **a**–**c** and **e**), and the maximum-to-minimum area ratio was calculated as 2.93 depending on the measurements on **a** and **b** (circled green lines). The dilated lumber veins could be recognized (**d**, **f**). IVC, inferior vena cava; LRV, left renal vein; RRA, right renal artery; CT, computed tomography. Long-shafted white arrows (without text), IVC; paired arrowheads, collateral veins

**Fig. 2:**
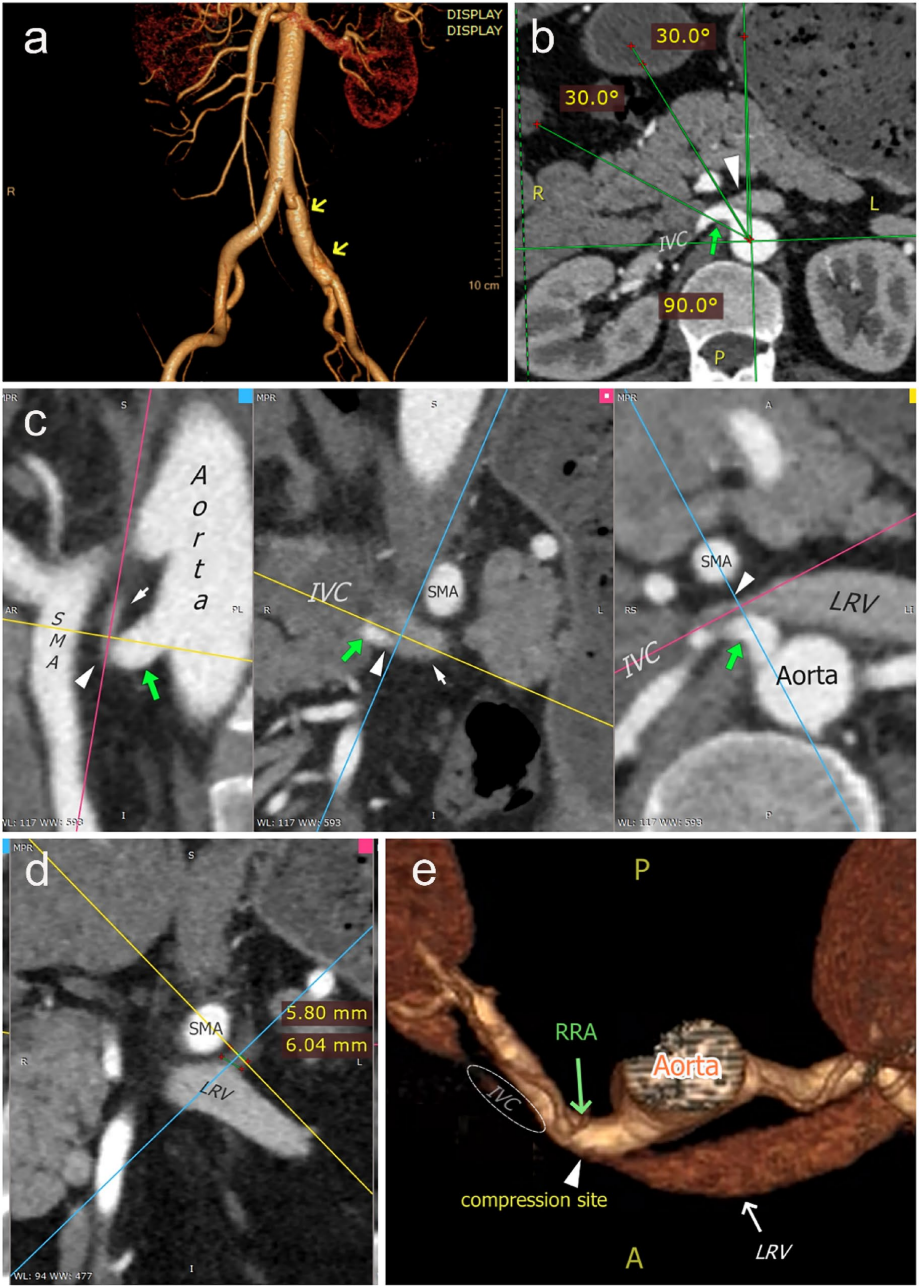
CT imaging showed a notable RRA-sourced LRV compression in a 48-year-old male with isolated iliac dissection (arrows in **a**). The initial segment of RRA (green arrows in **b**, **c**, and **e**), arising from an 11 o’clock direction (**b**), transversed behind and compressed (white arrowheads in **b, c**, and **e**) the ending segment of the LRV, where it joins the IVC. A dilated adrenal vein (the axes’ intersection in **d**) was visualized and measured. SMA, superior mesenteric artery; short-shafted white arrows (without text), LRV; other legends are the same as in Fig. 1

The arising direction of the initial segment of the RRA was measured in axial images, with a horizontal and a perpendicular straight line made through the center of the aorta where the RRA originated. Hour hand positions of 9–12 o’clock were projected and fused with the lined images, and the RRA arising directions were recorded as the hour hand positions (Fig. 1c, 2b).

The calibers of the collateral veins, i.e., the gonadal, ascending lumbar, and adrenal veins, were measured in the MPR planes perpendicular to the central axis of the vessel within the 2 cm length before they joined the LRV. The average values measured in millimeter were calculated and rounded after three measurements.

### Statistical analysis

Data processing was performed using SAS software version 9.4. Categorical variables were analyzed using Chi-square and Fisher’s exact tests, the Cochran–Mantel–Haenszel method for ordinal data, and the *t* test for numerical variables. All *p* values were two-sided, and values <0.05 were considered statistically significant.

## Results

### Baseline characteristics

After screening, 31 Renal and 25 Non-renal patients were included in the study. The mean age was 47.23 ± 6.43 (15–74) years and 62.24 ± 3.78 (41–81) years (*p* < 0.001), and the male sex ratio was 83.87% (26/31) and 68.00% (17/25) (*p* = 0.16) in the Renal and Non-renal groups, respectively. No LRV entrapment symptoms, including abdominal or lumbar pain, varicocele, or gross hematuria, were observed in the Renal patients (brief clinical data were presented in Online Resource 2). In the Non-renal group, the manifestations of neither corresponding peripheral arterial disease nor LRV hypertension were recorded. The diagnoses of the Non-renal patients were summarized in Online Resource 3.

### The rates of the LRV compression

The overall LRV entrapment rates by different adjacent structures were 38.71% (12/31) and 24.00% (6/25), and the rates of RRA-sourced LRV compression 22.58% (7/31) and 20.00% (5/25), and the rates of the superior mesenteric artery (SMA) compression 12.90% (4/31) and 4.00% (1/25) in the Renal (Fig. 1) and Non-renal (Fig. 2) groups, respectively, with no significance between the groups. Besides RRA and SMA, revealed sources of the LRV compression included the left renal artery (LRA, 3 Renal, 9.68%; 1 Non-renal, 4.00%), small intestine (2 Renal, 6.45%), and spleen artery(1 Non-renal, 4.00%). Complex compressions, namely, the compressions by another adjacent structure besides RRA, coincided in 5 cases (Online Resource 4; 2 by SMA besides RRA, Renal; 1 by RRA, SMA and LRA, Non-renal; 1 by RRA, SMA, LRA and duodenum, Renal; and 1 by spleen artery besides RRA, Non-renal). One renal case of duodenum-sourced LRV compression was considered to be related to the acute aortomesenteric angle (Online Resource 4), though no direct SMA contact was noticed in the patient. Therefore, the overall Renal incidence of SMA-sourced compression was calculated as 16.13% (5/31).

### Relevant parameters concerning the RRA-sourced compression

After excluding other anatomical compression factors, we analyzed the relationship between the RRA compression and multiple parameters in the Renal and Non-renal groups, with 22 patients in each group (Table 1). There was no gender or age difference between the Compressed and Non-compressed subgroups. The narrowest part of LRV had a high incidence at the ending segment before its confluence into IVC (77.27% Renal and 86.36% Non-renal, respectively, no significance). There was no statistical difference in the narrowest site classification between the groups and subgroups, and no cases were found with the narrowest site at the LRV Segment 1. The maximum-to-minimum ratios of the LRV transectional areas in the Compressed subgroups were significantly higher than that of the corresponding Non-compressed subgroups in both groups (3.09 ± 1.29 vs. 1.82 ± 0.23, *p* < 0.001; and 4.30 ± 2.65 vs. 2.12 ± 0.55, *p* = 0.006 in the Renal and Non-renal groups, respectively).

**Table 1:** Parameters related to the RRA-sourced LRV compression in the Renal and Non-renal groups

Parameters	Renal group (*n* = 22)			Non-renal group (*n* = 22)		
	Compressed	Non-compressed	*p*	Compressed	Non-compressed	*p*
*n* (/%)	4 (18.18)	18 (81.82)	–	3 (14.29)	19 (85.71)	–
Male (*n*, %)	2 (50.00)	16 (88.89)	0.14	2 (66.67)	14 (73.68)	1.00
Age (years)	46.75 ± 27.58	45.11 ± 9.68	0.88	56.33 ± 21.11	63.42 ± 4.32	0.22
Location of narrowest site of LRV (*n*, %)						
Segment 3	3 (75.00)	14 (77.78)	1.00	3 (100)	16 (84.21)	1.00
Segment 2	1 (25.00)	4 (22.22)		0	3 (16.67)	
Segment 1	0	0		0	0	
Angle^a^ (°)	52.23 ± 21.81	66.56 ± 9.55	0.18	57.33 ± 14.03	59.43 ± 6.48	0.80
Ratio^b^	3.09 ± 1.29	1.82 ± 0.23	<0.001	4.30 ± 2.65	2.12 ± 0.55	0.006
Diameters of the surrounding veins (average scores)						
Gonadal	3.00 ± 2.90	2.83 ± 0.71	0.84	3.33 ± 1.43	3.37 ± 0.67	0.97
Lumbar	6.00 ± 2.60	3.00 ± 1.04	0.01	3.33 ± 1.43	4.11 ± 0.94	0.51
Adrenal	3.25 ± 0.80	2.89 ± 0.41	0.42	3.33 ± 1.43	3.32 ± 0.43	0.97
Clockwise direction of the proximal right renal artery (*n*)						
9:00	0	1	0.37	0	2	0.86
9:30	0	3		1	2	
10:00	0	7		1	9	
10:30	3	5		0	4	
11:00	1	2		1	2	

^a^The aortomesenteric angle

^b^The maximum-to-minimum ratio of transectional LRV areas

The most common RRA arising orientation of the 44 patients were 10:00 and 10:30 (Table 1; Fig. 1c), with 17 (38.64%) and 12 (27.27%) cases, respectively, and with 3 (6.82%), 6 (13.64%) and 6 (13.64%) cases at 9 o’clock, 9:30, and 11 o’clock (Fig. 2b), respectively. There was no noticeable orientation difference between the groups and subgroups. Accessory right renal arteries were seen in 5 cases (4 Renal and 1 Non-renal), compressing the end of the LRV in 1 Renal patient.

The aortomesenteric angle was of no difference between the Renal and Non-renal groups and the Compressed and Non-compressed subgroups. Despite the diameter difference of the ascending lumbar veins between the Compressed and Non-compressed subgroups in the Renal group, there was no significant difference in gonadal or adrenal venous diameters between the subgroups within both groups (Table 1).

## Discussion

We found that the rates of LRV compression by RRA were remarkable in the two selected patient cohorts, both higher than that of the SMA-sourced compression detected in the corresponding groups. Larger distal LRV lumens were observed in the RRA-compressed subgroups than those in the non-compressed subgroups. However, no statistical differences between the groups or subgroups were revealed regarding the proximal RRA orientation, the classification of the narrowest LRV sites, and collateral venous dilatation.

To our knowledge, other LRV compression sources except SMA have scarcely been reported. Case reports revealing the RRA compression of LRV were sporadic before 2022, including 1 male and 3 females [8–11]. In 2022, Yoon studied 1475 NCP or nutcracker syndrome cases using sonography in a kidney-specialty referral clinic, revealing 50.6% of RRA-related LRV compression of the total cases [17]. Our study showed that RRA-sourced LRV compression was not rare but existed notably in two non-NCP patient groups. Similarly, the most frequently detected source of LRV entrapment in our study was RRA other than SMA.

The literature reported that the incidence of nutcracker syndrom tended to be higher in younger subjects [3]. The NCP rate detected in our study was much lower in peripheral arterial patients than in nephropathic patients, in which a significantly younger average age could be witnessed. In contrast, the rates of RRA-sourced LRV entrapment showed no such difference between groups.

Previous studies mostly measured the anteroposterior diameter of the LRV lumen on axial CT images to evaluate the changes in the LRV caliber caused by compression [4, 18–20]. The area measurement method adopted in the present study [21, 22] seems capable of evaluating the caliber sizes more accurately because it considers the possibility of a small anteroposterior distance with a disproportionately sizeable superoinferior distance (Figs. 1a, b, e, f, 2c), which is frequently seen in our patient cohorts (Online Resource 4). After excluding other sources of compression, we found that the distal segments of the LRVs compressed by RRAs have a significantly larger lumen area than those of non-compressed veins, suggesting that this compression may affect the venous drainage of LRV.

Of course, LRV pressure measurement under invasive methods would be of more meaning to judge venous hypertension despite the traumatic property of these investigations [18, 23, 24]. Regardless, we found in this study that the spatial resolution of the current widely used CT examination and the multiplanar analysis software had been validated to investigate the multiple relevant parameters of RRA compression from different angles, just as has been done in the NCP investigations [4, 14, 20, 25].

Polguj reported an 11 o’clock direction of proximal RRA, which compressed the LRV in a Caucasian female hospitalized due to choledocholithiasis [10]. In selected subjects, we found 8.9% (5/44) of patients have such an RRA arising direction (Fig. 2b). Notably, 68.2% (30/44) of the total cases had an arising direction of 10 o’clock or 10:30. In contrast, the proportion of those with a 9 o’clock arising direction accounts for only 6.82% (3/44). Though no corresponding significance between the Compressed and the Non-compressed subgroups was witnessed, a possible meaning of this finding is that the high proportion of the forward RRA course of its starting part might lay the ground for the direct contact with LRV and further compression of it by the RRA.

Previous studies divided LRV into two consecutive segments, lateral to and in front of the aorta [26], or the pre-aortic and post-aortic segments [19]. To study the compressing effect of RRA, we employed a trichotomy method, finding that the narrowest site of LRV in majority of cases were not at the aortomesenteric part, but at its very terminal of the LRV. Anatomically, RRA is usually closer to the end of LRV than SMA. We consider this, together with the high proportion of anteriorly rightward orientation of proximal RRA, explains the more frequent RRA-sourced compression detected than the NCP. Nevertheless, whether this observation correlates to the RRA compression must be clarified.

Kim reported the anatomic narrowing of the proximal left inferior pulmonary vein, which could be misinterpreted for possible venous stenosis [27]. Similarly, the narrowing of the ending segment of LRV, especially witnessed on axial CT, should not necessarily be interpreted as stenosis. On the other hand, a high incidence of terminal LRV narrowness should attract enough attention since it may theoretically undermine the therapeutic effects of operations such as stenting or replacing the aortomesenteric segment, which was frequently employed for treating LRV entrapment.

The LRV curves through the unique space surrounded by renal arteries, SMA, aorta, portal vein, and duodenum. Compared with the right renal vein, LRV is subjected to compressions by different surrounding anatomical structures other than SMA alone. Bushi and other teams reported LRV co-compression by duodenum and SMA [26, 28, 29]. LRA has also been reported as a source of compression for nutcracker syndrome [30]. In this study, we found that the RRA-sourced LRV compression coexisted with the compression by SMA, LRA, duodenum, or spleen artery in several cases. Given the controversies in the current diagnostic criteria and the uncertainty in the exact prevalence of NCP, we would consider the connotation of LRV compression requires more exploration. Further studies are needed to determine whether the CT findings in this study represent normal anatomical variations or the underlying pathogenic basis of the LRV entrapment syndrome.

This study has several limitations. Firstly, with CT as the sole investigating methodology, no pressure measurement or hemodynamic parameters were incorporated. Secondly, no association has been made between symptoms and diagnosis. Moreover, although comparable detection rates of the RRA compression were detected in the two cohorts, the small number of female subjects and sample size affected the generalizability of the study. Thirdly, because of the separate detection of all compression sources, the NCP rates calculated in this study can be lower and lack comparability with those reported in most literature. Lastly, we did find an LRV thrombosis case with membranous nephropathy in which an RRA-sourced LRV compression could be noticed (Online Resource 4). However, as a cross-sectional study, our finding does not indicate causality. Further studies involving larger sample sizes in different populations are warranted to establish the fact about its true incidence.

## Conclusions

Regarding LRV compression, RRA might be a more common compression source than SMA in selected Chinese patient populations and deserves careful differentiation when dealing with the syndromes of LRV entrapment. CT could be a practical tool to measure and diagnose the RRA compression from various angles. Further repetitive studies are needed to test if such a phenomenon prevails in different populations, including healthy individuals.

## Supplementary Information

Below is the link to the electronic supplementary material.

Supplementary file1 (PDF 196 KB)

Supplementary file2 (PDF 314 KB)

Supplementary file3 (PDF 320 KB)

Supplementary file4 (PDF 11224 KB)

Supplementary file5 (PDF 314 KB)

## Data Availability

The data that support the findings of this study are available from the corresponding author, Dr. Xuanyi Du, upon reasonable request.

## References

[1] Kurklinsky AK, Rooke TW (2010) Nutcracker phenomenon and nutcracker syndrome. Mayo Clin Proc 85:552–559. 10.4065/mcp.2009.0586 20511485 10.4065/mcp.2009.0586 PMC2878259

[2] Nastasi DR, Fraser AR, Williams AB, Bhamidi V (2022) A systematic review on nutcracker syndrome and proposed diagnostic algorithm. J Vasc Surg Venous Lymphat Disord 10:1410–1416. 10.1016/j.jvsv.2022.08.003 36007798 10.1016/j.jvsv.2022.08.003

[3] Ananthan K, Onida S, Davies AH (2017) Nutcracker syndrome: an update on current diagnostic criteria and management guidelines. Eur J Vasc Endovasc Surg 53:886–894. 10.1016/j.ejvs.2017.02.015 28356209 10.1016/j.ejvs.2017.02.015

[4] Poyraz AK, Firdolas F, Onur MR, Kocakoc E (2013) Evaluation of left renal vein entrapment using multidetector computed tomography. Acta Radiol 54:144–148. 10.1258/ar.2012.120355 23117197 10.1258/ar.2012.120355

[5] Yun SJ, Lee JM, Nam DH, Ryu JK, Lee SH (2016) Discriminating renal nutcracker syndrome from asymptomatic nutcracker phenomenon using multidetector computed tomography. Abdom Radiol (NY) 41:1580–1588. 10.1007/s00261-016-0717-8 27221972 10.1007/s00261-016-0717-8

[6] Grimm LJ, Engstrom BI, Nelson RC, Kim CY (2013) Incidental detection of nutcracker phenomenon on multidetector CT in an asymptomatic population: prevalence and associated findings. J Comput Assist Tomogr 37:415–418. 10.1097/rct.0b013e3182873235 23674014 10.1097/RCT.0b013e3182873235

[7] Unlu M, Orguc S, Serter S, Pekindil G, Pabuscu Y (2007) Anatomic and hemodynamic evaluation of renal venous flow in varicocele formation using color Doppler sonography with emphasis on renal vein entrapment syndrome. Scand J Urol Nephrol 41:42–46. 10.1080/00365590600796659 17366101 10.1080/00365590600796659

[8] Basile A, Tsetis D, Calcara G et al (2007) Nutcracker syndrome due to left renal vein compression by an aberrant right renal artery. Am J Kidney Dis 50:326–329. 10.1053/j.ajkd.2007.05.016 17660034 10.1053/j.ajkd.2007.05.016

[9] Stephens M, Ryan SK, Livsey R (2014) Unique nutcracker phenomenon involving the right renal artery and portal venous system. Case Rep Vasc Med 2014:579061. 10.1155/2014/579061 25097792 10.1155/2014/579061 PMC4102025

[10] Polguj M, Topol M, Majos A (2013) An unusual case of left venous renal entrapment syndrome: a new type of nutcracker phenomenon? Surg Radiol Anat 35:263–267. 10.1007/s00276-012-1027-7 23053120 10.1007/s00276-012-1027-7 PMC3604589

[11] Apruzzi L, Favia N, Bilman V, Ardita V, Chiesa R, Baccellieri D (2021) An uncommon variant of nutcracker syndrome secondary to left renal vein compression between the right renal artery and the proper hepatic artery. Ann Vasc Surg 77:352.e313-352.e317. 10.1016/j.avsg.2021.06.007 10.1016/j.avsg.2021.06.007 34455053

[12] Zigman A, Yazbeck S, Emil S, Nguyen L (2000) Renal vein thrombosis: a 10-year review. J Pediatr Surg 35:1540–1542. 10.1053/jpsu.2000.18302 11083418 10.1053/jpsu.2000.18302

[13] Li SJ, Guo JZ, Zuo K et al (2012) Thromboembolic complications in membranous nephropathy patients with nephrotic syndrome—a prospective study. Thromb Res 130:501–505. 10.1016/j.thromres.2012.04.015 22633211 10.1016/j.thromres.2012.04.015

[14] Zhang LJ, Zhang Z, Li SJ et al (2014) Pulmonary embolism and renal vein thrombosis in patients with nephrotic syndrome: prospective evaluation of prevalence and risk factors with CT. Radiology 273:897–906. 10.1148/radiol.14140121 25072187 10.1148/radiol.14140121

[15] von Elm E, Altman DG, Egger M, Pocock SJ, Gøtzsche PC, Vandenbroucke JP (2014) The strengthening the reporting of observational studies in epidemiology (STROBE) statement: guidelines for reporting observational studies. Int J Surg 12:1495–1499. 10.1016/j.ijsu.2014.07.013 25046131 10.1016/j.ijsu.2014.07.013

[16] Arthurs OJ, Mehta U, Set PA (2012) Nutcracker and SMA syndromes: What is the normal SMA angle in children? Eur J Radiol 81:e854–e861. 10.1016/j.ejrad.2012.04.010 22579528 10.1016/j.ejrad.2012.04.010

[17] Yoon T, Kim SH, Kang E, Kim S (2022) Nutcracker phenomenon and syndrome may be more prevalent than previously thought. Korean J Radiol 23:1112–1114. 10.3348/kjr.2022.0617 36305049 10.3348/kjr.2022.0617 PMC9614295

[18] Kim KW, Cho JY, Kim SH et al (2011) Diagnostic value of computed tomographic findings of nutcracker syndrome: correlation with renal venography and renocaval pressure gradients. Eur J Radiol 80:648–654. 10.1016/j.ejrad.2010.08.044 20869828 10.1016/j.ejrad.2010.08.044

[19] Zerin JM, Hernandez RJ, Sedman AB, Kelsch RC (1991) “Dilatation” of the left renal vein on computed tomography in children: a normal variant. Pediatr Radiol 21:267–269. 10.1007/bf02018620 1870922 10.1007/BF02018620

[20] Góes AMO, Araújo RS, Furlaneto IP, Vieira WB (2020) Compression of left renal vein and left common iliac vein on CT scans: how often are they detected? J Vasc Bras 19:e20190121. 10.1590/1677-5449.190121 34178065 10.1590/1677-5449.190121 PMC8202167

[21] Caussin C, Daoud B, Ghostine S et al (2005) Comparison of lumens of intermediate coronary stenosis using 16-slice computed tomography versus intravascular ultrasound. Am J Cardiol 96:524–528. 10.1016/j.amjcard.2005.04.013 16098305 10.1016/j.amjcard.2005.04.013

[22] Yuan XP, Bach D, Skanes A, Drangova M (2004) Assessment of intra- and interobserver variability of pulmonary vein measurements from CT angiography. Acad Radiol 11:1211–1218. 10.1016/j.acra.2004.07.016 15561567 10.1016/j.acra.2004.07.016

[23] Nishimura Y, Fushiki M, Yoshida M et al (1986) Left renal vein hypertension in patients with left renal bleeding of unknown origin. Radiology 160:663–667. 10.1148/radiology.160.3.3737903 3737903 10.1148/radiology.160.3.3737903

[24] Debucquois A, Salomon du Mont L, Bertho W, Kaladji A, Hartung O, Rinckenbach S (2021) Current results of left gonadal vein transposition to treat nutcracker syndrome. J Vasc Surg Venous Lymphat Disord 9:1504–1509. 10.1016/j.jvsv.2021.03.003 33737260 10.1016/j.jvsv.2021.03.003

[25] Moselewski F, Ropers D, Pohle K et al (2004) Comparison of measurement of cross-sectional coronary atherosclerotic plaque and vessel areas by 16-slice multidetector computed tomography versus intravascular ultrasound. Am J Cardiol 94:1294–1297. 10.1016/j.amjcard.2004.07.117 15541250 10.1016/j.amjcard.2004.07.117

[26] Buschi AJ, Harrison RB, Norman A et al (1980) Distended left renal vein: CT/sonographic normal variant. AJR Am J Roentgenol 135:339–342. 10.2214/ajr.135.2.339 6773339 10.2214/ajr.135.2.339

[27] Kim YH, Marom EM, Herndon JE, II, McAdams HP (2005) Pulmonary vein diameter, cross-sectional area, and shape: CT analysis. Radiology 235:43–49; discussion 49–50. 10.1148/radiol.2351032106 10.1148/radiol.2351032106 15731371

[28] Diab S, Hayek F (2020) Combined superior mesenteric artery syndrome and nutcracker syndrome in a young patient: a case report and review of the literature. Am J Case Rep 21:619. 10.12659/ajcr.922619 10.12659/AJCR.922619 PMC7440741 32772039

[29] Heidbreder R (2018) Co-occurring superior mesenteric artery syndrome and nutcracker syndrome requiring Roux-en-Y duodenojejunostomy and left renal vein transposition: a case report and review of the literature. J Med Case Rep 12:214. 10.1186/s13256-018-1743-7 30081961 10.1186/s13256-018-1743-7 PMC6091179

[30] Sawant DA, Moore TF (2015) An unusual course of segmental renal artery displays a rare case of hilar nutcracker phenomenon. Case Rep Med 2015:249015. 10.1155/2015/249015 26448765 10.1155/2015/249015 PMC4584064

