# Supplementary material for: A new notable compression source of left renal vein entrapment: right renal artery
Source: World J Urol. 2024 May 29;42(1):360. doi: 10.1007/s00345-024-05053-7 (PMC11136829; doi:10.1007/s00345-024-05053-7)
Supplement: Supplementary file 1 — Supplementary file1 (PDF 196 KB) [file 345_2024_5053_MOESM1_ESM.pdf]

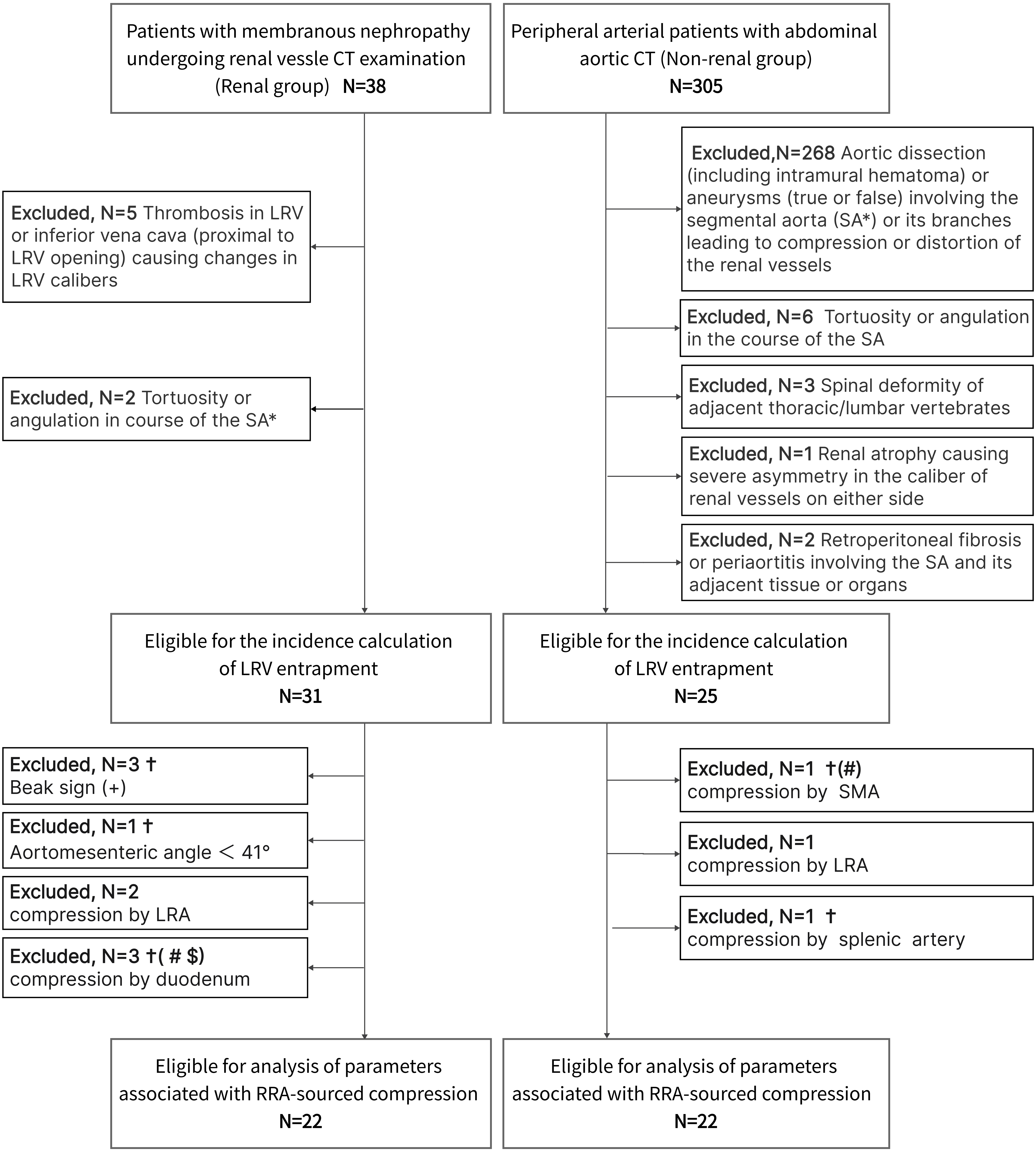

Supplement Material 1 Flowchart of the two patient cohorts with membranous nephropathy and peripheral arterial diseases between November 2018 and March 2023. CT, computed tomography; LRV, left renal vein; LRA, left renal artery; RRA, right renal artery; SMA, superior mesenteric artery; *SA, the segmental aorta between the distal edge of the celiac artery opening and the horizontal line corresponding to the lower pole of the lower kidney;†, with one case with RRA-sourced compression; †(#), the case was complex compression by RRA, LRA besides SMA; †(# $), with one complex compression by RRA, LRA and SMA, besides duodenum.

Article title: A New Notable Compression Source of Left Renal Vein Entrapment: the Right Renal Artery.
Journal name: *World Journal of Urology*
Authors: Zhanfeng Sun, M.D., Haitao Wang, M.D., Huijie Jiang, Yongbin Shen, Ziming Shi, Qingxiao Wang, Han Wang, Weiliang Jiang, Xuanyi Du, M.D*.
Corresponding author: Prof. Dr. Xuanyi Du, M.D., the Second Affiliated Hospital of Harbin Medical University, Harbin, Heilongjiang, China; Email address: dxy_shennei@126.com
