# Supplementary material for: A new notable compression source of left renal vein entrapment: right renal artery
Source: World J Urol. 2024 May 29;42(1):360. doi: 10.1007/s00345-024-05053-7 (PMC11136829; doi:10.1007/s00345-024-05053-7)
Supplement: Supplementary file 2 — Supplementary file2 (PDF 314 KB) [file 345_2024_5053_MOESM2_ESM.pdf]

Supplement Material 2 Clinical data of the included patients with membranous nephropathy.

Patients (n=31)	
Mean age (yr, range)	47.23 (15-74)
Male (n, %)	26 (83.87%)
Acute course within one month (n, %)	10 (32.3%)
Steroid treatment before admission (n, %)	3 (9.7%)
Abdominal or lumbar pain (n, %)	0
Varicocele or gross hematuria (n, %)	0
Microscopic haematuria† (n, %)	10 (32.3%)
Lower limb edema (n, %)	29 (93.6%)
Plasma albumin (g/L)	25.57±9.68
Serum creatinine (umol/L)	104.20±33.30

†, before biopsy if applicable.

Article title: A New Notable Compression Source of Left Renal Vein Entrapment: the Right Renal Artery.

Journal name: *World Journal of Urology*

Authors: Zhanfeng Sun, M.D., Haitao Wang, M.D., Huijie Jiang, Yongbin Shen, Ziming Shi, Qingxiao Wang, Han Wang, Weiliang Jiang, Xuanyi Du, M.D*.

Corresponding author: Prof. Dr. Xuanyi Du, M.D., the Second Affiliated Hospital of Harbin Medical University, Harbin, Heilongjiang, China; Email address: dxy_shennei@126.com
