# Supplementary material for: A new notable compression source of left renal vein entrapment: right renal artery
Source: World J Urol. 2024 May 29;42(1):360. doi: 10.1007/s00345-024-05053-7 (PMC11136829; doi:10.1007/s00345-024-05053-7)
Supplement: Supplementary file 3 — Supplementary file3 (PDF 320 KB) [file 345_2024_5053_MOESM3_ESM.pdf]

Diagnosis	Location of lesion	Descriptions or remarks on the inclusion	n
Aneurysms	Aortic arch	With IMH within the proximal descending aorta in one.	3
	Descending aorta	With a tortuous spleen artery in one case.	4
	Abdominal aorta	All was distant from the renal vessels; 1 complicated with pseudoaneurysm of the right common iliac artery and a mass about 2.5×2×2cm^3 in volume in the right lower lung.	3
	Splenic artery	Close to the hilus of the spleen and not squeezing the renal vessels	1
Dissections or IMHs	Descending aorta	All lesions terminated above the celiac trunk, complicated with arch PAU in one, with true aneurysm in the arch in another.	7
	Abdominal aorta	Minor infrarenal dissection, over 10mm away below the opening of the lower renal vessel	1
	SMA	Localized middle segmental SMA dissection, the initial portion not affected	1
	Common iliac artery	With a splenic mass (6.0×6.4×4.3cm^3), distant from the studied area in one case.	2
PAUs	Aortic arch	Lesions limited to the arch, with IMH	1
	Descending aorta	Localized lesion only	2
Arterial occlusion	Infrainguinal arteries	With localized abdominal aortic dissection, over 10mm away below the opening of lower renal vessel	1
**Total**			25

Supplement Material 3 Imaging diagnosis of the included Non-renal subjects. IMH, intramural hematoma; PAU, penetrating aortic ulcer; SMA, superior mesenteric artery.

Article title: A New Notable Compression Source of Left Renal Vein Entrapment: the Right Renal Artery.

Journal name: *World Journal of Urology*

Authors: Zhanfeng Sun, M.D., Haitao Wang, M.D., Huijie Jiang, Yongbin Shen, Ziming Shi, Qingxiao Wang, Han Wang, Weiliang Jiang, Xuanyi Du, M.D*.

Corresponding author: Prof. Dr. Xuanyi Du, M.D., the Second Affiliated Hospital of Harbin Medical University, Harbin, Heilongjiang, China; Email address: dxy_shennei@126.com
