# Supplementary figures and images for: A new notable compression source of left renal vein entrapment: right renal artery
Source: World J Urol. 2024 May 29;42(1):360. doi: 10.1007/s00345-024-05053-7 (PMC11136829; doi:10.1007/s00345-024-05053-7)

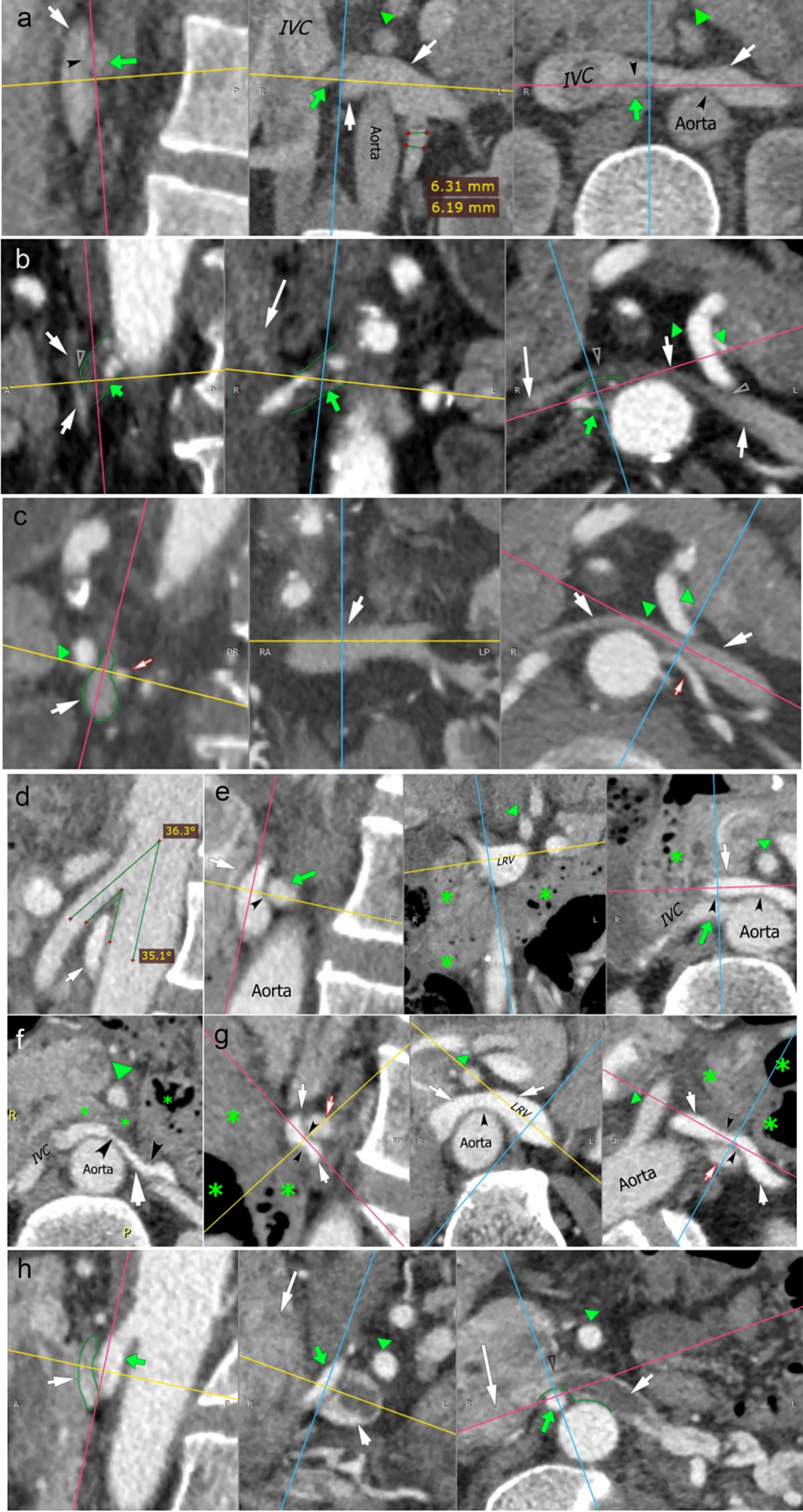

Supplement: Supplementary file 4 — Supplementary file4 (PDF 11224 KB) [file 345_2024_5053_MOESM4_ESM.pdf]
