# Supplementary material for: A new notable compression source of left renal vein entrapment: right renal artery
Source: World J Urol. 2024 May 29;42(1):360. doi: 10.1007/s00345-024-05053-7 (PMC11136829; doi:10.1007/s00345-024-05053-7)
Supplement: Supplementary file 5 — Supplementary file5 (PDF 314 KB) [file 345_2024_5053_MOESM5_ESM.pdf]

Supplement Material 4 The complex RRA-sourced compression cases and a thrombosed LRV compressed by RRA. **a**, compression by RRA plus SMA in a 30-year-old female with membranous nephropathy; **b&c**, compression by RRA plus splenic artery in a 66-year-old male with a pseudoaneurysm in the thoracic aorta; **d-g**, compression by RRA, SMA, duodenum, and LRA in a 60-year-old male with membranous nephropathy, complicated by chronic portal venous thrombosis and incomplete ileus. **h**, chronic LRV thrombosis in a 57-year-old male with membranous nephropathy. IVC, inferior vena cava; LRA, left renal artery; LRV, left renal vein; RRA, right renal artery; green arrows, the proximal RRA; white arrows (without text), short-shafted, LRV and long-shafted, IVC; red-edged white arrows, LRA; green arrowheads, SMA; paired green arrowheads, splenic artery; black or white arrowheads, compression sites; paired white arrowheads or measured straight lines, dilated collateral veins; asterisks, small intestine; green curved lines, outer borders of a vessel.

Article title: A New Notable Compression Source of Left Renal Vein Entrapment: the Right Renal Artery.

Journal name: *World Journal of Urology*

Authors: Zhanfeng Sun, M.D., Haitao Wang, M.D., Huijie Jiang, Yongbin Shen, Ziming Shi, Qingxiao Wang, Han Wang, Weiliang Jiang, Xuanyi Du, M.D*.

Corresponding author: Prof. Dr. Xuanyi Du, M.D., the Second Affiliated Hospital of Harbin Medical University, Harbin, Heilongjiang, China; Email address: dxy_shennei@126.com
